# Keel bone fractures are more prevalent in White Leghorn hens than in Red Jungle fowl hens—A pilot study

**DOI:** 10.1371/journal.pone.0255234

**Published:** 2021-07-27

**Authors:** Käthe Elise Kittelsen, Pall Gretarsson, Per Jensen, Jens Peter Christensen, Ingrid Toftaker, Randi Oppermann Moe, Guro Vasdal

**Affiliations:** 1 Animalia- The Norwegian Meat and Poultry Research Centre, Oslo, Norway; 2 Faculty of Veterinary Medicine, NMBU—Norwegian University of Life Sciences, Oslo, Norway; 3 AVIAN Behavioural Genomics and Physiology Group, IFM Biology, Linkoping University, Linkoping, Sweden; 4 Department of Veterinary & Animal Sciences, University of Copenhagen, Copenhagen, Denmark; Tokat Gaziosmanpasa Universitesi, TURKEY

## Abstract

Fractures and deviations to the keel bone are common in commercial laying hens, with reported variations in occurrence across strains and breeds. The aetiology is not fully understood, however, modern genetics and selection for efficient egg production has been claimed to be important factors for the keel bone fractures. To explore this further, we investigated keel bones from two different breeds, representing different degrees of selection for egg production: Red jungle fowl (n = 82), and White Leghorn (n = 32), where the latter is a selected laying breed which is the origin for many modern laying hen hybrids. Keel bones from a total of 116 birds, 53 hens and 63 roosters, were examined by necropsy at 80 weeks of age. All birds were raised in modified aviaries in the same holding facility. Overall, 24.5% of the hens had one or more fractures to the keel, with a difference in the prevalence between hens from the two breeds (p<0.01): 10% (95% CI: 3.7–24%) in the Red Jungle fowl hens and 69% (95% CI: 37–90%) in the White Leghorn hens. No roosters, regardless of breed, had keel bone fractures. Mild to moderate keel bone deviations were present in 54% (95% CI: 25–80%) of the hens and 4.7% (95% CI: 0.5–30%) of the roosters, all White Leghorns.

## Introduction

Animal husbandry for food production has changed enormously in the Western world after World War II [1, 2]. The overall aim has been an efficient and reliable food production. For the egg industry, this focus has resulted in the highly productive hybrids used in commercial egg production today [2].

Modern laying hens descend from the Red jungle fowl (*Gallus gallus*) in South East Asia [3, 4], domesticated more than 9000 years ago [4]. Throughout the centuries this bird has been spread worldwide and selected for different phenotypic qualities, resulting in a large number of breeds with a variety of traits. Around 1955 a rapid transformation in the egg industry took place, when a multitude of breeds were replaced by a few hybrids [5, 6]. Nearly all new hybrids derived from a handful of layer breeds, including the White Leghorn (*Gallus gallus domesticus*) [2, 7]. Since then, the White Leghorn has been used as the genetic origin of highly productive egg laying hybrids, due to its efficient egg production and high feed conversion rate [8]. Today, many modern layers are commercial strains of the White Leghorn.

Modern selection programs for commercial laying hen hybrids have had a strong emphasis on high egg production and efficient feed conversion [2]. Domestic poultry are more fertile, produce more eggs and mature earlier than their wild ancestor [4, 8, 9]. The Red jungle fowl in the wild will lay 4–6 eggs each year [10, 11]. However, Red jungle fowl hens in captivity may produce more; two eggs per week in the season (April to August) is reported by Schütz et al [8]. Around the year 1900, a selected egg-laying hen produced 83 eggs yearly [12]. Today, modern layers typically produce close to 350 eggs per hen up to 75 weeks of age (WOA) [2]. In addition to focusing on the overall number of eggs, selection has also focused on an early onset of lay. A commercial layer hen starts to lay the first eggs at 16 WOA [13]. In comparison, the Red jungle fowl hen in the wild is reportedly around 42 WOA old when she starts to lay the first eggs [9], while captive Red jungle fowl hens are approximately 25 WOA old [8].

It has been speculated that genetic selection for increased production may cause skeletal health issues, such as keel bone damage (KBD) [14, 15]. KBD is comprised of two different conditions affecting the keel: keel bone deviations and keel bone fractures (KBF). Keel bone deviations are a morphological change to the keel defined as bone with an abnormal shaped form, not resulting from a fracture [16], but is linked to other factors such as pressure on the keel during perching [17]. KBF are characterized as sharp bends or fragmented sections of the keel bone [16]. Prevalence of KBF in commercial production are alarmingly high; it can affect up to 97% of hens within a flock end of lay in loose housed systems [18, 19]. In addition to the high prevalence, KBF is likely associated with pain [20, 21], making it one of the most serious welfare issues the laying hen industry is facing today [17, 22]. The causative factors for KBF in commercial laying hens are not clear, it is likely multifactorial and different breeds and strains exhibit different KBF prevalence Egg production has been suggested as one of the contributing factors. There are different aspects of egg production that may be linked to KBF including a high number of eggs produced per hen [15], early onset of lay [23] and an immature keel during top egg production [24]. The negative impact of egg production on KBF development is supported by the fact that fractures are not observed in roosters [25, 26] or in hens treated with hormones to prevent egg laying [27].

Only one previous publication has investigated keel bones from Red jungle fowls, which reports that KBF were less prevalent in Red jungle fowl hens housed in a research facility [25] as compared to reported prevalences in commercial layer hybrids. The prevalence of KBF in Red jungle fowl hens compared to more selected breeds housed under identical conditions will give important information on the genetic component in KBF. To shed light on the effect of genetics and selection for egg production on the occurrence of KBF in layers, the aim of the current study was to investigate prevalence of KBF in Red Jungle Fowls and White Leghorns housed in identical conditions, representing different degrees of selection and egg production.

## Material and methods

### Birds and management

The study group consisted of 116 birds: 82 Red Jungle fowls; 42 roosters and 40 hens, and 34 White Leghorns; 21 roosters and 13 hens. The birds were selected from a larger population kept at the University of Linköping. The Red Jungle fowls were not purebred; they originated from two different zoo populations originally crossed to increase genetic variability. The two crossed populations were from Copenhagen Zoo and Götala research station (SLU). The White Leghorn line used in this study is called SLU13 and is a non-commercial breed selected for egg production. SLU13 is maintained at the Swedish University of Agricultural Sciences. All birds in the study population were of the same age and they were examined at the same time. The birds were raised in pens consisting of 30 animals, breeds and sexes kept in separate pens within the same building. The lack of individual tagging made it unfortunately not possible to know which pen the 116 birds originated from, but they were distributed across pens along with other birds in the holding facility. The birds were housed in a modified multitier aviary system (Vencomatic Bolegg Terrace from Vencomatic Group, Eersel, The Netherlands), divided into pens of 3 x 3 x 3 meters. The modification consisted of a segmentation of the aviary, so that it could fit into the pens. The stocking density was lower than EU regulations [30]. In addition, all birds had unlimited access to a 3 x 3 x 2-meter outdoor area. The aviary consisted of two tiers; the first was 100 cm above the floor and the second tier 220 cm above the floor. The floor level was covered with wood shaving as dustbathing material. The first tier had metal wired floor. All levels were equipped with round perches of metal, 35 mm in diameter. Nest boxes were situated in the first tier. The nipple drinkers were located on the first tier and on the floor level. Automatic feeding chains in the first tier provided the birds with commercial ad libitum feed (supplier: Lantmännen AB, 104 25 Stockholm).

The study was approved by the Linköping council for ethical licencing of animal experiments, ethical permit no 14916–2018.

### Keel bone examination

**Fig 1:**
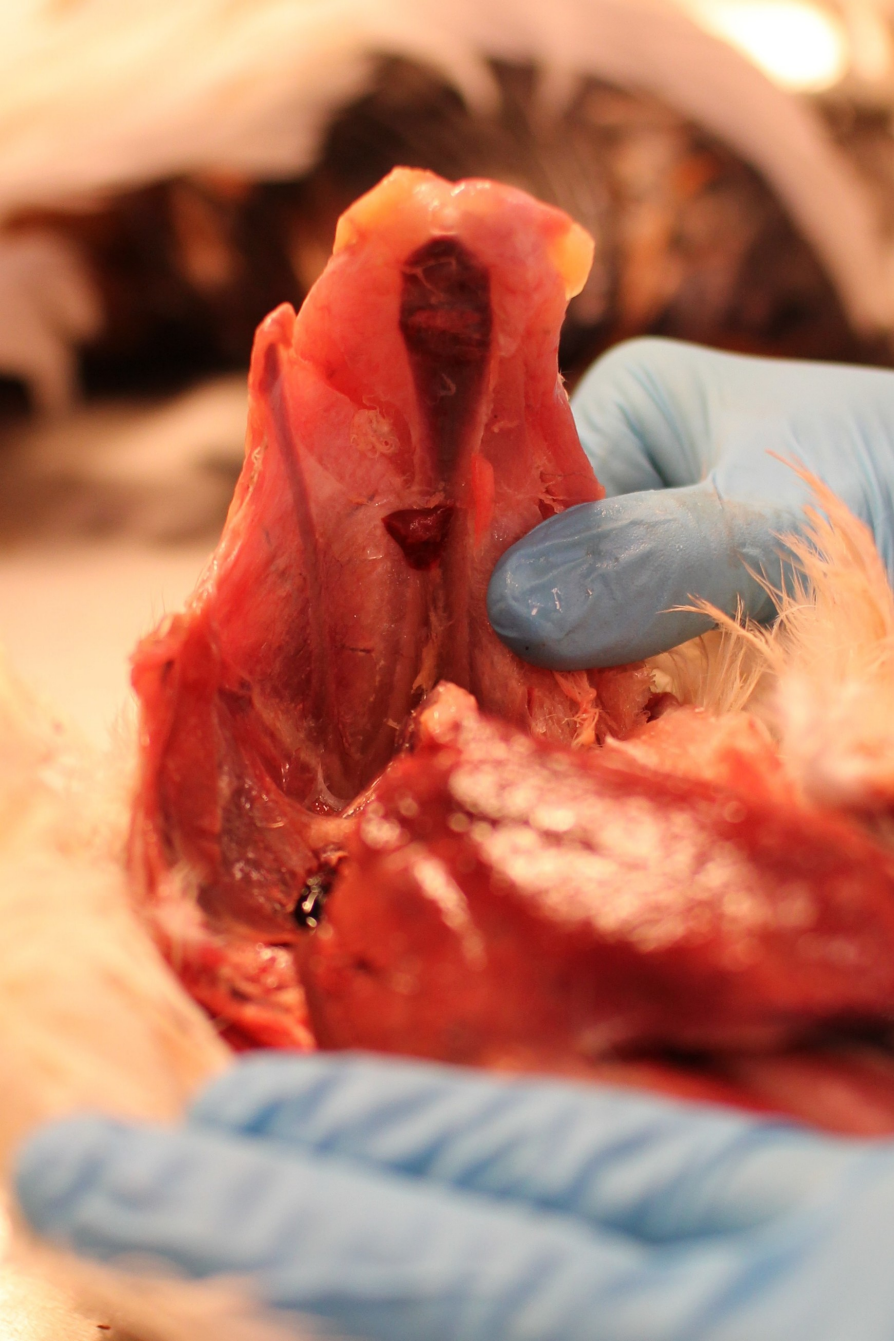
Examination of the dorsal side of the keel bone during necropsy.

All birds were culled by cervical dislocation and decapitation at the age of 80 weeks and then shipped frozen, express overnight, from the University of Linköping, Sweden, to the Norwegian University of Life Sciences, Oslo, Norway for examination. Evaluation of the keel bones was performed on thawed birds by an experienced poultry veterinarian, trained by the EU Cost Keel Bone Action’s training school. The keel bone was first palpated for factures and deformations according to the Simplified Keel Assessment Protocol [16]. External palpation was followed up by necropsy, including inspection of the dorsal side of the keel bone to avoid underestimation due to palpation only. See Fig 1 for examination of the dorsal side of the keel bone. The keel bone was classified as having a deformation during palpation if there was an abnormal shaped form either in the transverse or sagittal plane, not resulting from a fracture. The keel bone was classified as having a fracture during necropsy if there was a visible or palpable callus formation on either side of the keel bone or a thin, dark, slightly protruding line on the dorsal side, which is a fracture without callus formation. Additionally, during necropsy, fractures were classified according to which part of the keel the fracture was located; cranial, middle, or caudal part, along with the total number of fractures per anatomical site.

### Statistics

Occurrence of fractures and deviations were presented as proportions with confidence intervals. For the hens, Fishers exact test was used to test the difference in fracture prevalence between Red Jungle fowls and White Leghorns, as well as calculating a confidence interval for the risk difference. A cut-off of p< 0.05 was considered as statistically significant. Data entry was performed in Excel, and data was later transferred to Stata [28] for data management and statistical analysis.

## Results

Regardless of breed, none of the 63 roosters had KBF. For all hens, 24.5% had one or more fractures to the keel. The palpation scoring underestimated the true prevalence of KBF in this study; only 4 of the 13 KBF in the hens could be detected during palpation. The majority of the fractures were observed during necropsy with inspection of the dorsal side of the keel bone. The prevalence of having at least one fracture differed between hens from the two breeds. It was 69% (95% CI: 37–90%) in White Leghorn hens and 10% (95% CI: 3.7–24%) in the Red Jungle fowl hens, resulting in a risk difference of 59% (95% CI: 32–86%; p<0.001). The prevalence of keel bone fractures and deviations are presented in Table 1. One of the White Leghorn hens exhibited two keel bone fractures, all the other hens had just one KBF. All fractures were located dorsally on the caudal tip of the keel bone.

**Table 1:** Prevalence and 95% confidence intervals of keel bone fractures and keel bone deviations in 82 Red Jungle fowls and 34 White Leghorns based on necropsy.

	Red Jungle fowl n = 82						White Leghorn n = 34					
	Hens n = 40			Roosters n = 42			Hens n = 13			Roosters n = 21		
	n	%	95% CI	n	%	95% CI	n	%	95% CI	n	%	95% CI
**No fractures**	36	90%	75–97%	42	100%	-	4	31%	10–63%	21	100%	-
**One or more fractures**	4	10%	3.7–24%	0	0%	-	9	69%	37–90%	0	0%	-
**No deviations**	40	100%	-	42	100%	-	6	46%	20–75%	20	95%	70–100%
**Mild or moderate deviations**	0	0%	-	0	0%	-	7	54%	25–80%	1	4.7%	0.5–30%

## Discussion

To the best of our knowledge, this is the first study to compare keel bone fractures and deformations in Red Jungle fowls and White Leghorns, housed in the same environment and system. Although this is a pilot study with a limited study population, the results give important and new insight; there was a considerable difference in the prevalence of both KBF and keel bone deformations between the breeds housed in the same facility. Furthermore, no KBF were detected in the roosters, regardless of breed and KBF were present in Red Jungle fowl hens, although far fewer than observed in the White leghorns.

### KBF and breed

The prevalence of KBF was significantly lower for Red jungle fowl hens compared to White Leghorn hens. The prevalence in the Red Jungle fowl hens is in agreement with a recent study on KBD in the same breed [25]. The finding in the White Leghorn hens is comparable to the results presented in studies conducted on modern, commercial laying hen strains [11, 16]. A difference between breeds and between lines and hybrids have been documented in several previous studies [14, 29, 30], and the present results are in line with existing literature.

Although the causative factors behind the observed KBF in the present study is unknown, our results support findings in several previous papers which suggest that genetics and selection for egg production are important factors [31, 32]. There are several important production parameters that may be linked to KBF; early onset of lay is one aspect [23]. The White Leghorn hybrid in this study is reported to have an onset of lay at 19.9 weeks, while the Red Jungle fowl hen strain used have an onset of lay five weeks later (24.9 weeks) [8]. Exactly why an early onset of lay may be negative for the keel bone, is unclear. One theory is late ossification of the keel [33]; the ossification of the caudal part of the keel is not completed until 30–40 weeks of age [34, 35], indicating that the modern hen start laying eggs 15–20 weeks before the keel bone is fully mature. The caudal part of the keel is also the most common place for fractures [18, 24]. This is in line with the results in the current study: all fractures were located at caudal third of the keel, regardless of breed. The immature bone in an ossification zone is weaker than mature bone and may therefore be more prone to fractures [36]. The prolonged stress period caused by an early onset of lay combined with a late ossification might therefore increase the risk of fractures. Thøfner et al. found that the fracture lines on the dorsal side of the keel bone in layer hens resembles green stick fractures [24]. Green stick fractures in children are associated with ossification zones [37], which further points to the late ossification as causative factor for KBF. Age for complete ossification of the keel bone in different breeds and hybrids have not been published previously, thus we do not know if this development differs between birds with a different genetic background. This warrants further studies. Other relevant factors could be conflicting needs of minerals for growth and eggshell formation at a young age.

In addition to an earlier onset of lay, the White Leghorn hybrid in this study is reported to lay a higher number of eggs and heavier eggs than the Red Jungle fowl hen strain [8]. The mean egg number per week for the White Leghorns was 6.0, while the same number for the Red Jungle fowl hens was 2.6 [8]. This finding is in line with results from Jung et al [38], who found a positive correlation between high egg production and KBF prevalence. Schütz et al found the mean egg weight, in gram, for the White Leghorn hybrid to be 57.5. For the Red Jungle fowl hens in the same study, the mean egg weight was 23 grams [8]. Egg size at onset of lay may also be a risk factor for the development of KBF, as shown in recent a risk analysis (Christensen, personal communication, unpublished).

There are striking differences in the reported production results between the Red Jungle fowl strain and the White Leghorn hybrid used in this study. It must be noted that the production results are collected from a previous study with the same breeds at the same holding facility. These results may not be comparable to the current flock, but they indicate a difference in the egg production between the breeds. The lack of individual level production data in the present study, rendered it impossible to disentangle single causative factors. However, the difference in both KBF and egg production between the breeds points in the same direction as several published studies. A major strength to the present study is that all birds were housed in the same facility, meaning confounding related to housing or management was considered negligible. A limitation of the study is that the lack of individual animal tagging made it impossible to account for nesting of animals in different pens in the analysis, thus a possible pen-effect should be noted as a potential source of bias.

Keel bone deformations were detected in 54% of the White Leghorn hens and in one White Leghorn rooster. It must be noted that all deformations were mild or moderate, no severe deviations were observed. In comparison, none of the Red Jungle fowl hens or roosters had keel bone deformations. This is in contrast to a previous investigation of Red Jungle fowls from the same facility, where 83% of the hens and 5.9% of the roosters were diagnosed with a keel bone deviation [25]. The reason for the difference in keel bone deviations between the two studies may be the age of the birds; in the present study the birds were culled at the age of 80 weeks compared to 112 weeks in the previous study. Keel bone deviations and fractures are assumed to have different aetiology. The deviations of the keel bone are believed to be caused by extended perching and a long-term pressure on the keel bone [39]. A study by Stratmann et al showed that soft perches reduced the prevalence of keel bone deviations versus hard perching material [40]. The causative factors for the deviation difference between the two breeds need to be investigated further in future studies.

### KBF and Red Jungle fowls

KBF in Red Jungle fowls have been examined just once, in a pilot study [25]. The results in that study showed that 10% of the hens had KBF, which matches the prevalence found in current study. This indicates that keel bone fractures will occur in this breed when housed under modern poultry management. It must also be noted that previous production results for Red Jungle fowl hens in the same facility indicates more fertile hens that produce much more eggs and mature earlier than their wild counterparts [9–11]. The KBF prevalence for wild non-selected Red Jungle fowl hens in natural surroundings remains unknown. Investigation of these hens should be carried out in the future to examine if KBF occurs in Red Jungle fowl hens with a different genetic background and in their natural habitat.

### KBF and sex

None of the roosters in this study, regardless of breed, had keel bone fractures. This is in line with previous examinations of roosters [25, 26, 30]. Interestingly, laying hens treated with hormones to inhibit egg production do not develop keel bone fractures [27, 32]. This may be due to the effect of estrogen; high estrogen levels during sexual maturity affects bone formation so medullary bone is built in the long bones, which is important as a calcium reservoir. In addition, the high estrogen levels cease the formation of cortical bone and thus weakened structural bones cannot be repaired [41]. In addition, the mobilisation of calcium form structural bone renders the bone weakened. Accordingly, Eusemann et al. (2020) found that hormone-treated hens with inhibited egg production showed a higher radiographic density in the keel bone compared to egg laying hens, in addition to no keel bone fractures [32]. This may indicate that either sexual steroids, egg laying or a combination of these influence the risk for fractures.

## Conclusion

Of the two breeds housed in the same facility, 24.5% of the hens had one or more fractures to the keel, with significantly more fractures in the more selected White Leghorn hens. No roosters, regardless of breed, had keel bone fractures. Even though the sample size is small, the findings from this study emphasize the importance of genetics and breed as risk factors for the development of keel bone fractures. Of the Red Jungle fowl hens, 10% had a keel bone fracture, indicating that such fractures also occur in breeds with less artificial selection, although far lower than reported in commercial layer lines. The difference in KBF prevalence between the two breeds, representing different degrees of domestication and egg production, suggests that genetic selection is a promising approach to improve the keel bone health in layers.

## Supporting information

S1 Data (XLSX)
